# The Relationship between the Urinary Cadmium Concentration and Cause-Specific Mortality in Subjects without Severe Renal Damage: A 35-Year Follow-Up Study in a Cadmium-Polluted Area of Japan

**DOI:** 10.3390/ijerph18157747

**Published:** 2021-07-21

**Authors:** Masaru Sakurai, Yasushi Suwazono, Muneko Nishijo, Kazuhiro Nogawa, Yuuka Watanabe, Kazuka Yoneda, Masao Ishizaki, Yuko Morikawa, Teruhiko Kido, Hideaki Nakagawa

**Affiliations:** 1 Department of Social and Environmental Medicine, Kanazawa Medical University, Ishikawa 920-0293, Japan; db8-2152@kanazawa-med.ac.jp (K.Y.); issa1@kanazawa-med.ac.jp (M.I.); hnakagaw@kanazawa-med.ac.jp (H.N.); 2 Health Evaluation Center, Kanazawa Medical University, Ishikawa 920-0293, Japan; 3 Department of Occupation and Environmental Medicine, Graduate School of Medicine, Chiba University, Chiba 260-8670, Japan; suwa@faculty.chiba-u.jp (Y.S.); nogawa@chiba-u.jp (K.N.); watanabe155@chiba-u.jp (Y.W.); 4 Department of Epidemiology and Public Health, Kanazawa Medical University, Ishikawa 920-0293, Japan; ni-koei@kanazawa-med.ac.jp; 5 School of Nursing, Kanazawa Medical University, Ishikawa 920-0293, Japan; ymjr@kanazawa-med.ac.jp; 6 School of Health Sciences, College of Medical, Pharmaceutical and Health Sciences, Kanazawa University, Ishikawa 920-0942, Japan; tkido@staff.kanazawa-u.ac.jp

**Keywords:** urinary cadmium, renal tubular dysfunction, mortality, cohort study

## Abstract

We evaluated the association between urinary cadmium concentration (uCd, μg/g Cr) and risk of cause-specific mortality according to urinary β2-microglobulin (MG) concentration. Participants were 1383 male and 1700 female inhabitants of the Cd-polluted Kakehashi River basin. The uCd and β2-MG were evaluated in a survey in 1981–1982, where those participants were followed-up over 35 years later. Among the participants with a urinary β2-MG < 1000, the hazard ratios (HRs) (95% confidence interval) for mortality were significantly higher in those with a uCd of ≥10.0 compared with <5.0 for cardiovascular disease [HR 1.92 (1.08–3.40) for men, 1.71 (1.07–2.71) for women], pneumonia or influenza [2.10 (1.10–4.00) for men, 2.22 (1.17–4.19) for women], and digestive diseases [for men; 3.81 (1.49–9.74)]. The uCd was significantly associated with mortality from heart failure in women and digestive diseases in men, after adjustment for other causes of death using the Fine and Gray competing risk regression model. For participants with a urinary β2-MG of ≥1000, no significant association was observed between uCd and any major cause of death. In the absence of kidney damage, Cd may increase the risk of death from cardiovascular disease, pneumonia, and digestive diseases.

## 1. Introduction

**Figure 1:**
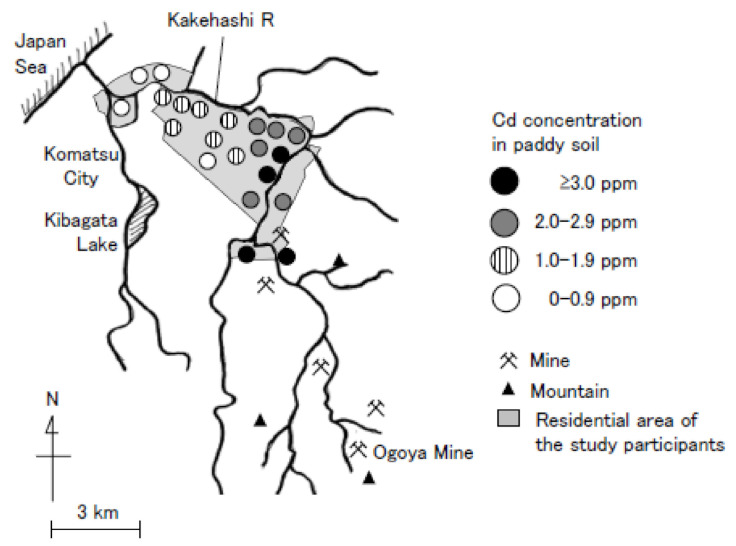
Location of copper mines and the cadmium (Cd)-polluted area of the Kakehashi River basin. (Source. Ishikawa Prefecture, 1975).

The Kakehashi River basin of Ishikawa Prefecture, Japan is polluted by cadmium (Cd) because of upstream copper mining that began in the 1600s (Figure 1). We followed subjects living in this area since a baseline survey was conducted in 1981–1982 and reported higher mortality rates in subjects with greater Cd exposure in follow-ups after 9, 15, and 22 years [1,2,3,4,5,6]. At the 15- and 22-year follow-ups, the subjects with higher urinary Cd concentrations exhibited increased rates of all-cause mortality as well as mortality due to renal disease and heart failure [4,6]. Recently, we evaluated the risks of all-cause and cause-specific mortalities in inhabitants of the Kakehashi River basin, in a follow-up after 25 years, and reported that the urinary concentrations of Cd and beta 2-microglobulin (β2-MG) (a marker of renal tubular dysfunction induced by Cd exposure) were associated with higher risks of all-cause mortality and mortality due to renal disease [7,8]. We reported that [9] the standard mortality rates (SMRs) for all-cause of participants with a urinary β2-MG of ≥1000 mg/g Cr were significantly higher (127 for men and 146 for women) than for the Japanese general population. By contrast, the SMRs of those with a urinary β2-MG < 1000 mg/g Cr were not significantly higher compared to the Japanese general population. However, the impact of Cd exposure on mortality in those without severe renal dysfunction was not evaluated.

Higher Cd exposure is reportedly associated with mortality from certain causes, including cancer [10,11], cardiovascular disease [12,13,14], pneumonia or influenza [15], and renal disease [2,4,6,9,16]. However, some causes of death, such as cancer and pneumonia, were not associated with Cd exposure levels in inhabitants of the Cd-polluted area of the Kakehashi River basin [4,6,9]. The urinary Cd concentration was markedly higher in studies of Cd-polluted areas of Japan compared with Western countries, and this may have affected the results. The kidney is a target of external Cd exposure and renal tubular dysfunction is the most prevalent adverse health effect induced by Cd exposure. The high risk of renal disease caused by high Cd exposure may confound the risks of other diseases in inhabitants of Cd-polluted areas of Japan. Therefore, it is necessary to analyze the relationship between the Cd level and the risk of cause or cause-specific mortality after excluding participants with severe renal dysfunction, or to examine the risk of cause-specific mortality while considering the competing risks. A competing risk is an outcome that precludes the occurrence of the event of interest. The urinary Cd level was reported to be associated with several causes of death, including cancer, cardiovascular disease, and renal disease, and it is important to consider the competing risks when evaluating the association between the urinary Cd level and a specific cause of mortality. The Fine and Gray competing risk regression model can be used to evaluate the risk of death from a specific disease by considering competing mortality events [17].

In this follow-up study of inhabitants of the Kakehashi River basin, 35 years later, we evaluated the association between urinary Cd concentration and mortality risk according to the presence of renal tubular dysfunction to clarify the risk of cause-specific mortality with consideration of the competing risks, using the Fine and Gray competing risk regression model.

## 2. Materials and Methods

### 2.1. Study Population

Detailed information on the study population has been published previously [4,7,8,18]. In brief, a total of 3178 inhabitants (1424 men and 1754 women) of the Cd-polluted Kakehashi River basin who participated in the health impact survey in 1981–1982 were targeted in the present study. The health impact survey was conducted by the government of Ishikawa Prefecture and targeted all inhabitants of the area aged over 50 years at that time; the participation rate was 91%. Urine samples were collected early in the morning. In the 1981–1982 survey, the urinary Cd level was measured by atomic absorption spectrophotometry and the urinary β2-MG level by radioimmunoassay (Phadebas β2-microtest; Pharmacia Diagnostics AB, Uppsala, Sweden). The values were corrected by reference to the urinary creatinine (Cr) levels measured by the Jaffe method.

### 2.2. Follow-Up

The survival status of the participants was confirmed in 1991, 1998, 2003, and 2016. In this study, 35-year follow-up data were analyzed. Participants were followed-up from the day of the initial examination during the health impact survey of 1981–1982 up until November 2016. With the cooperation of the Prefectural Public Health Office and City Municipal Office, we confirmed the survival status (alive or dead) and place of residence (still residing in or moved out of the target area) of the participants. For those confirmed dead, the date of death was ascertained from death certificates. We confirmed the causes of death by matching the data to those of the National Vital Statistics database, using the area, sex, date of birth, and date of death as key codes. The causes of death were classified according to the International Statistical Classification of Diseases and Related Health Problems, 9th revision (ICD-9) until 1994 and ICD-10 from 1995.

Of the 3178 potential participants, 95 were excluded for the following reasons: missing urinary Cd concentration data (n = 56), lost to follow-up (e.g., due to moving out of the target area; n = 15), or cause of death unconfirmable because the data did not match those of the National Vital Statistics database (n = 24). Finally, 3083 participants (1383 men and 1700 women) were enrolled in the present study.

### 2.3. Statistical Analysis

Participants were divided into two groups according to the extent of baseline renal damage, as assessed by the urinary β2-MG concentration (<1000 or ≥1000 μg/g Cr). The risks of mortality were calculated separately for men and women. Age-adjusted hazard ratios (HRs) for all-cause and cause-specific mortalities were calculated using the Cox proportional hazards model according to the urinary Cd concentration (<5.0 [reference], 5.0–9.9, and ≥10.0 μg/g Cr). These analyses were performed using the Statistical Package for the Social Sciences (ver. 26.0; IBM Corporation, Armonk, NY, USA). Age-adjusted risk ratios (RRs) for cause-specific mortality for each urinary Cd concentration range were calculated using the Fine and Gray competing risk regression model, with other mortality events considered as the competing risks. The Fine and Gray model was performed using the ‘cmprsk’ package of R statistical software (ver. 3.1.0; R Core Team, Vienna, Austria). A *p*-value < 0.05 was considered to indicate statistical significance.

The Ethics Committee of Kanazawa Medical University approved the present study (approval no. 212, 2014).

## 3. Results

At the baseline examination, the mean (standard deviation) age was 63.1 (9.0) years for men and 63.8 (9.2) years for women. The geometric mean (95% confidence interval [CI]) urinary Cd concentration (μg/g Cr) was 4.6 (1.4–15.2) in men and 7.2 (2.2–23.2) in women. The urinary β2-MG concentration was less than 1000 μg/g Cr in 86.1% of men and 81.5% of women. Table 1 shows the numbers of participants, mortalities, and person-years of follow-up, as well as mortality rates, according to sex. The mortality rate (/1000 person-years) was 43.1 for men and 31.0 for women and was lower in participants with a urinary β2-MG concentration of <1000 μg/g Cr compared with ≥1000 μg/g Cr (both men and women) (Table 1).

Table 2 shows the causes of death among men and women according to the extent of renal damage. The most common cause of death in men without severe renal damage was a malignant neoplasm (22.9%). Circulatory disease was the principal cause of death in men with severe renal damage (35.4%) and in women with (35.0%) and without (19.8%) severe renal damage. The rate of mortality from a malignant neoplasm was similar between the participants for those with and without severe renal damage. On the other hand, more subjects with severe renal damage died from circulatory disease.

Table 3 shows the risks of all-cause and cancer-specific mortalities according to the urinary Cd concentration in participants without severe renal damage. In men, the risk of all-cause mortality tended to be higher in those with a urinary Cd concentration of ≥10.0 μg/g Cr compared with <5.0 μg/g Cr, but the difference was not statistically significant. In women, the risk of all-cause mortality was associated with the urinary Cd concentration (*p* for trend = 0.035). Cox proportional hazard models revealed significant associations between the urinary Cd concentration and mortality from any malignant neoplasm or organ-specific neoplasms in both men and women. The results were similar after adjusting for the competing risks using the Fine and Gray regression model.

Table 4 shows the risks of mortality from circulatory disease according to the urinary Cd concentration in participants without severe renal damage. In men, the HR for mortality from cardiovascular disease was significantly higher in those with a urinary Cd concentration of ≥10.0 μg/g Cr compared with <5.0 μg/g Cr, but the difference was not statistically significant after adjusting for the competing risks. In women, the risk of heart failure was significantly associated with urinary Cd concentration (*p* for trend = 0.004) in the Cox regression model and remained statistically significant even after adjusting for the competing risks using the Fine and Gray regression model (*p* for trend = 0.038).

Table 5 shows the risks of mortality from non-cancer/non-circulatory diseases according to the urinary Cd concentration in participants without severe renal damage. The Cox regression model showed that those with a urinary Cd concentration of ≥10.0 μg/g Cr were at significantly higher risk of pneumonia compared with those with a urinary Cd concentration of <5.0 μg/g Cr (in both men and women). The urinary Cd concentration was significantly and linearly associated with mortality from pneumonia in women (p for trend = 0.014). However, this association was weakened after adjusting for the competing risks using the Fine and Gray regression model. In men, the urinary Cd concentration was significantly associated with mortality from digestive diseases in both the Cox regression model (*p* for trend = 0.014) and the Fine and Gray model (*p* for trend = 0.028). Of the various digestive diseases, the major cause of death was liver cirrhosis, and the risk of such death tended to be higher in those with a urinary Cd concentration of 5.0–9.9 or ≥10.0 μg/g Cr compared with <5.0 μg/g Cr, but the difference was not statistically significant. In women, the risk of mortality from external causes was significantly associated with the urinary Cd concentration in the Cox regression model (*p* for trend = 0.044), and the association tended to remain after adjusting for the competing risks using the Fine and Gray regression model (*p* for trend = 0.054).

For participants with a urinary β2-MG concentration of ≥1000 μg/g Cr, no significant association was observed between any major cause of death and the urinary Cd concentration (Table 6).

## 4. Discussion

In this follow-up study of inhabitants of the Kakehashi River basin, 35 years later, we evaluated the association between the urinary Cd level and mortality risk in participants without severe renal tubular dysfunction. The results showed that the urinary Cd concentration was significantly associated with mortality from heart failure in women and from diseases of the digestive system in men, even after adjustment for other causes of death using the Fine and Gray competing risk regression model. We previously reported that the urinary β2-MG concentration was associated with a high risk of mortality from renal disease, but not from other causes, in both men and women of the present cohort, as revealed by a competing risk model [8]. The kidney is a target of external Cd, and renal tubular dysfunction is the most prevalent adverse health effect induced by Cd exposure. Thus, renal disease may be a common cause of death in individuals with renal tubular dysfunction, and it may be difficult to evaluate the risk of non-renal mortality using the renal tubular function test, even though this reflects the extent of Cd exposure. Therefore, in the present study, we investigated the association between the urinary Cd concentration, which is a direct index of Cd exposure, and the cause-specific mortalities of participants without severe renal tubular dysfunction.

Among the participants without severe renal tubular dysfunction, those with high urinary Cd concentrations exhibited a significantly higher HR for mortality from cardiovascular disease, but this association was weakened after adjustment for other causes of death. Among cardiovascular diseases, a urinary Cd level of ≥10.0 μg/g Cr tended to be associated with a higher risk of death from ischemic heart disease in men, and with a significantly higher risk of death from heart failure in women. The risk of death from heart failure in women remained after adjustment for renal disease and other causes of death. These results indicate that Cd exposure is particularly strongly associated with cardiovascular disease in participants without severe renal tubular dysfunction associated with Cd exposure. Some previous studies have suggested that Cd exposure may increase the risk of death from cardiovascular disease [12,13,14], whereas studies conducted in the UK [19] and Belgium did not [20]. An association between Cd exposure and increased cardiovascular disease mortality is biologically plausible. The experimental evidence supports a role for Cd in atherosclerosis, as Cd may increase inflammation [21] and endothelial oxidative stress [22,23]. Furthermore, higher Cd exposure is associated with a higher LDL cholesterol level [24], lower HDL cholesterol level [25], and higher levels of inflammatory markers, such as C-reactive protein [24] and the plasma-soluble urokinase plasminogen activator receptor [26]. The dyslipidemia and systemic inflammation caused by Cd exposure may lead to cardiovascular disease among inhabitants of Cd-polluted areas. Thus, Cd exposure may increase the risk of cardiovascular disease independently of its effects on the kidneys, and the results of this study indicate that participants without severe renal dysfunction may be more likely to die from cardiovascular disease.

We found that the HR for mortality from pneumonia or influenza in those with a urinary Cd level of ≥10.0 μg/g Cr was approximately 2.0, which was significantly higher than the HR in participants with a concentration of <5.0 μg/g Cr. However, this association was weakened after adjustment for other causes of death. A recent study in a general US population showed that those with a high-urinary Cd level were at increased risk of death from pneumonia or influenza [15]. Such associations were also observed in non-smokers, suggesting that Cd may increase the risk of pneumonia regardless of the source (tobacco or contaminated food). In another study, the urinary Cd concentration was associated with reduced lung function, regardless of smoking status [27], and the reduced lung function resulted in worse outcomes of pulmonary infectious diseases. Cd accumulates in human lung tissue [28] and may affect the progression and prognosis of lung disease. For example, Cd potentiates cytokine production and lung inflammation [29], impairs macrophage-mediated immune function [30], and disrupts tight junction integrity in the epithelial tissue of human airways at the air–liquid interface [31]. Cd also binds to the sulfhydryl groups of thiols and thus interferes with the antioxidant defense system, altering the redox balance and creating oxidative stress [32].

Among men without severe renal tubular dysfunction, those with a high urinary Cd concentration exhibited a significantly higher HR for mortality from digestive diseases. This association was significant even after adjusting for other causes of death. The numbers of participants who died from gastric and duodenal ulcers, and from ileus and intestinal obstructions, were too small to allow for an evaluation of the association with the renal Cd concentration. The HR for death from liver cirrhosis in participants with a urinary Cd level of 5.0–9.9 or ≥10.0 μg/g Cr was approximately 2.0; however, this association was not significant. A previous study suggested that the Cd concentration is higher in the liver than in any other organ of an itai-itai disease group, and hepatic fibrosis was significantly more common in that group compared with controls [33]. Cd accumulation in the liver due to chronic exposure promotes liver fibrosis and may increase the risk of death from liver cirrhosis. An association between urinary Cd concentration and death from digestive diseases was found only in men. Men generally drink more than women, and the effects of alcohol may influence the association between the Cd concentration and death from cirrhosis. However, the amount of alcohol consumed is not known, and thus any effect of alcohol could not be evaluated.

Cd is a causative agent of several cancers, including lung [11], breast [34,35], prostate [36], and renal cancers [37]. However, cancer mortality was not related to Cd exposure in inhabitants of the Cd-polluted area of the Kakehashi River basin in several studies [4,6,9], including the present study. Such an association was also not detected in the Jinzu River basin [38], although one study did report a high cancer mortality rate in this basin [16], particularly among women with renal damage [39]. The urinary Cd concentrations were markedly higher in studies conducted in Cd-polluted areas of Japan compared with Western countries. Relatively low Cd exposure may increase the risk of cancer-specific mortality, but fa urther increase in Cd exposure may not further increase the risk. Alternatively, the difference may be attributable to the different routes of exposure; workers are exposed to Cd principally via the airways, whereas those living in Cd-polluted areas of Japan are mostly exposed orally.

Of participants without severe renal tubular dysfunction, Cd exposure may affect mortality from cardiovascular disease, pneumonia and influenza in both men and women, as well as digestive diseases in men. On the other hand, no significant association was observed between Cd exposure and any cause-specific mortality in participants with severe renal tubular dysfunction. We previously reported that the urinary concentration of β2-MG, a marker of renal tubular dysfunction, was associated with all-cause mortality as well as renal disease-specific mortality in inhabitants of a Cd-polluted area [8]. Renal tubular dysfunction may directly increase the risk of death from renal disease regardless of Cd exposure, suggesting that Cd is not associated with death in participants with severe renal damage. In addition, the small number of participants with severe tubular dysfunction may have reduced this statistical accuracy.

The strengths of this study include the relatively large sample size and long follow-up period (over 35 years), which enabled evaluation of cause-specific mortality. However, when evaluating the association between Cd concentration and cause-specific death, any target cause of death may be affected by a competing outcome (i.e., another cause of death). We accounted for competing risks using the Fine and Gray competing risk regression model. This study also had certain limitations. Firstly, the only confounding factor used in the statistical model was age. Other factors such as the presence of hypertension and other lifestyle-related diseases, smoking status, alcohol intake, and other lifestyle factors may affect the relationship between Cd concentration and mortality. We lacked data on these possible confounders. In addition, the number of participants with a urinary β2-MG concentration of ≥1000 μg/g Cr was small, which explains why the association between all-cause mortality and the urinary Cd concentration was not significant in participants with severe renal damage. Furthermore, the Cd concentration was evaluated only once in the 1981–1982 survey. By 1988, rice paddy restoration was underway in this area and Cd exposure may have changed during the follow-up period. However, our findings indicate that the long-term prognosis of participants living in the Cd-polluted area is unfavorable.

## 5. Conclusions

The 35 years of follow-up results from inhabitants of the Cd-polluted area of the Kakehashi River basin indicated that a high urinary Cd concentration was associated with higher risks of all-cause mortality and of mortality caused by cardiovascular disease, pneumonia, or digestive diseases among participants without severe renal tubular dysfunction. Even in the absence of kidney damage, Cd can induce death from causes other than kidney disease.

## Figures and Tables

**Table 1:** Numbers of participants and person-years of follow-up according to the urinary β2-MG concentration.

	Men		Women	
	β2-MG (μg/g Cr)		β2-MG (μg/g Cr)	
	<1000	≥1000	<1000	≥1000
Number of participants	1191	192	1386	314
Number of deaths during the follow-up period (%)	905 (76.0)	184 (95.8)	816 (58.9)	279 (88.9)
Total person-years of observation	23,102	2142	4184	31,187
Mortality rate (/1000 person-years)	39.2	85.9	26.2	66.7
Mean observed years per person	19.4	11.2	22.5	13.3

**Table 2:** Cause of mortality by sex and the urinary β2-MG concentration.

	Men, n (%)		Women, n (%)	
	β2-MG (μg/g Cr)		β2-MG (μg/g Cr)	
	<1000	≥1000	<1000	≥1000
Malignant neoplasms	273 (22.9)	38 (19.8)	193 (13.9)	38 (12.1)
Esophagus	4 (0.3)	1 (0.5)	1 (0.1)	3 (1.0)
Stomach	49 (4.1)	8 (4.2)	28 (2.0)	4 (1.3)
Colon, rectum	31 (2.6)	4 (2.1)	28 (2.0)	7 (2.2)
Liver	33 (2.8)	1 (0.5)	17 (1.2)	0 (0.0)
Pancreas	25 (2.1)	5 (2.6)	30 (2.2)	3 (1.0)
Lung	62 (5.2)	10 (5.2)	22 (1.6)	7 (2.2)
Prostate	16 (1.3)	1 (0.5)	0 (0.0)	0 (0.0)
Breast	0 (0.0)	0 (0.0)	8 (0.6)	3 (1.0)
Uterus	0 (0.0)	0 (0.0)	2 (0.1)	0 (0.0)
Ovary	0 (0.0)	0 (0.0)	6 (0.4)	1 (0.3)
Bladder	6 (0.5)	2 (1.0)	3 (0.2)	2 (0.6)
Kidney	4 (0.3)	0 (0.0)	1 (0.1)	0 (0.0)
Lymphatic or hematopoietic tissue	13 (1.1)	4 (2.1)	16 (1.2)	1 (0.3)
Endocrine, nutritional, and metabolic diseases	14 (1.2)	2 (1.0)	18 (1.3)	7 (2.2)
Diabetes	8 (0.7)	2 (1.0)	12 (0.9)	4 (1.3)
Circulatory disease	251 (21.1)	68 (35.4)	275 (19.8)	110 (35.0)
Cardiovascular disease	124 (10.4)	31 (16.1)	141 (10.2)	61 (19.4)
Ischemic heart disease	36 (3.0)	9 (4.7)	31 (2.2)	14 (4.5)
Heart failure	66 (5.5)	15 (7.8)	84 (6.1)	38 (12.1)
Cerebrovascular disease	113 (9.5)	32 (16.7)	128 (9.2)	46 (14.6)
Cerebral hemorrhage	23 (1.9)	2 (1.0)	28 (2.0)	7 (2.2)
Cerebral infarction	58 (4.9)	7 (3.6)	64 (4.6)	7 (2.2)
Subarachnoidal hemorrhage	4 (0.3)	0 (0.0)	14 (1.0)	2 (0.6)
Diseases of the respiratory system	162 (13.6)	24 (12.5)	113 (8.2)	22 (7.0)
Pneumonia and influenza	96 (8.1)	12 (6.3)	76 (5.5)	16 (5.1)
Diseases of the digestive system	33 (2.8)	9 (4.7)	33 (2.4)	17 (5.4)
Gastric and duodenal ulcer	1 (0.1)	3 (1.6)	1 (0.1)	2 (0.6)
Ileus and intestinal obstruction	3 (0.3)	3 (1.6)	5 (0.4)	2 (0.6)
Liver cirrhosis	11 (0.9)	2 (1.0)	8 (0.6)	3 (1.0)
Kidney and urinary tract diseases	22 (1.8)	13 (6.8)	20 (1.4)	15 (4.8)
Renal disease	0 (0.0)	2 (1.0)	2 (0.1)	2 (0.6)
Renal failure	16 (1.3)	9 (4.7)	12 (0.9)	12 (3.8)
Senility	39 (3.3)	14 (7.3)	71 (5.1)	44 (14.0)
External causes of mortality	53 (4.4)	6 (3.1)	37 (2.7)	9 (2.9)
Toxic effects of cadmium	1 (0.1)	0 (0.0)	2 (0.1)	0 (0.0)
β2-MG: β2-microglobulin.				

**Table 3:** Risk of all-cause mortality and cancer-specific mortality according to the urinary Cd concentration in the participants with a urinary β2-MG level less than 1000 μg/g Cr.

		Urinary Cd Concentration (μg/g Cr)			*p* for Trend
		<5.0	5.0–9.9	≥10.0	
Men					
All-cause mortality	n	550	292	63	
	Mortality rate (/1000 PY)	37.9	41.1	42.8	
	HR (95%CI)	1.00 (reference)	1.08 (0.94–1.24)	1.23 (0.95–1.60)	0.092
Malignant neoplasm	n	171	88	14	
	Mortality rate (/1000 PY)	11.8	12.4	9.5	
	HR (95%CI)	1.00 (reference)	1.06 (0.82–1.37)	0.87 (0.51–1.51)	0.95
	RR (95%CI)	1.00 (reference)	1.00 (0.78–1.30)	0.70 (0.41–1.20)	0.37
Stomach	n	27	20	2	
	Mortality rate (/1000 PY)	1.9	2.8	1.4	
	HR (95%CI)	1.00 (reference)	1.53 (0.86–2.73)	0.78 (0.19–3.27)	0.47
	RR (95%CI)	1.00 (reference)	1.45 (0.82–2.58)	0.66 (0.16–2.74)	0.64
Colon, rectum	n	21	7	3	
	Mortality rate (/1000 PY)	1.4	0.99	2.0	
	HR (95%CI)	1.00 (reference)	0.66 (0.28–1.54)	1.43 (0.43–1.48)	0.85
	RR (95%CI)	1.00 (reference)	0.65 (0.28–1.51)	1.29 (0.39–4.28)	0.78
Liver	n	22	10	1	
	Mortality rate (/1000 PY)	1.5	1.4	0.68	
	HR (95%CI)	1.00 (reference)	0.96 (0.45–2.02)	0.49 (0.07–3.65)	0.58
	RR (95%CI)	1.00 (reference)	0.90 (0.43–1.90)	0.41 (0.06–3.02)	0.40
Pancreas	n	17	6	2	
	Mortality rate (/1000 PY)	1.2	0.84	1.4	
	HR (95%CI)	1.00 (reference)	0.73 (0.29–1.86)	1.31 (0.30–5.70)	0.86
	RR (95%CI)	1.00 (reference)	0.68 (0.27–1.73)	1.07 (0.25–4.65)	0.69
Lung	n	34	27	1	
	Mortality rate (/1000 PY)	2.3	3.8	0.68	
	HR (95%CI)	1.00 (reference)	1.61 (0.97–2.67)	0.31 (0.04–2.28)	0.62
	RR (95%CI)	1.00 (reference)	1.57 (0.95–2.61)	0.26 (0.04–1.89)	0.81
Prostate	n	13	3	0	
	Mortality rate (/1000 PY)	0.90	0.42	0	
	HR (95%CI)	1.00 (reference)	0.49 (0.14–1.73)	-	0.42
	RR (95%CI)	1.00 (reference)	0.45 (0.13–1.57)		0.08
Lymphatic or hematopoietic tissue	n	9	3	1	
	Mortality rate (/1000 PY)	0.62	0.42	0.68	
	HR (95%CI)	1.00 (reference)	0.65 (0.18–2.42)	1.19 (0.15–9.41)	0.77
	RR (95%CI)	1.00 (reference)	0.64 (0.17–2.36)	0.99 (0.13–7.88)	0.70
Women					
All-cause mortality	n	228	404	184	
	Mortality rate (/1000 PY)	25.1	26.7	26.4	
	HR (95%CI)	1.00 (reference)	1.19 (1.01–1.40)	1.22 (1.00–1.49)	0.035
Malignant neoplasm	n	58	93	42	
	Mortality rate (/1000 PY)	6.4	6.1	6.0	
	HR (95%CI)	1.00 (reference)	0.97 (0.70–1.35)	0.97 (0.65–1.45)	0.89
	RR (95%CI)	1.00 (reference)	0.91 (0.66–1.27)	0.90 (0.61–1.35)	0.61
Stomach	n	9	11	8	
	Mortality rate (/1000 PY)	0.99	0.73	1.15	
	HR (95%CI)	1.00 (reference)	0.75 (0.31–1.80)	1.19 (0.46–3.09)	0.64
	RR (95%CI)	1.00 (reference)	0.70 (0.29–1.70)	1.14 (0.44–2.92)	0.79
Colon, rectum	n	8	15	5	
	Mortality rate (/1000 PY)	0.88	0.99	0.72	
	HR (95%CI)	1.00 (reference)	1.16 (0.49–2.75)	0.87 (0.28–2.68)	0.87
	RR (95%CI)	1.00 (reference)	1.09 (0.46–2.58)	0.80 (0.26–2.45)	0.73
Liver	n	5	10	2	
	Mortality rate (/1000 PY)	0.55	0.66	0.29	
	HR (95%CI)	1.00 (reference)	1.20 (0.41–3.51)	0.53 (0.10–2.75)	0.55
	RR (95%CI)	1.00 (reference)	1.14 (0.39–3.33)	0.50 (0.10–2.57)	0.43
Pancreas	n	9	13	8	
	Mortality rate (/1000 PY)	0.99	0.86	1.15	
	HR (95%CI)	1.00 (reference)	0.87 (0.37–2.04)	1.19 (0.46–3.08)	0.76
	RR (95%CI)	1.00 (reference)	0.83 (0.36–1.95)	1.14 (0.44–2.96)	0.83
Lung	n	7	12	3	
	Mortality rate (/1000 PY)	0.77	0.79	0.43	
	HR (95%CI)	1.00 (reference)	1.11 (0.44–2.85)	0.62 (0.16–2.43)	0.58
	RR (95%CI)	1.00 (reference)	1.00 (0.39–2.53)	0.56 (0.14–2.22)	0.43
Lymphatic or hematopoietic tissue	n	5	7	4	
	Mortality rate (/1000 PY)	0.55	0.46	0.57	
	HR (95%CI)	1.00 (reference)	0.85 (0.27–2.67)	1.05 (0.28–3.93)	0.97
	RR (95%CI)	1.00 (reference)	0.81 (0.26–2.56)	1.03 (0.28–3.81)	1.00

β2-MG: β2-microglobulin; Cd: cadmium; n, number of deaths; PY, person-years; HR, age-adjusted hazard ratio calculated using the Cox proportional hazards model; RR, age-adjusted risk ratio calculated by the Fine and Gray competing risk regression model; CI, confidence interval.

**Table 4:** Risk of mortality from circulatory disease according to the urinary Cd concentration in the participants with a urinary β2-MG level less than 1000 μg/g Cr.

		Urinary Cd Concentration (μg/g Cr)			*p* for Trend
		<5.0	5.0–9.9	≥10.0	
Men					
Circulatory disease	n	158	74	19	
	Mortality rate (/1000 PY)	10.9	10.4	12.9	
	HR (95%CI)	1.00 (reference)	0.94 (0.71–1.24)	1.29 (0.80–2.09)	0.67
	RR (95%CI)	1.00 (reference)	0.86 (0.65–1.15)	1.11 (0.68–1.82)	0.73
Cardiovascular disease	n	77	33	14	
	Mortality rate (/1000 PY)	5.3	4.6	9.5	
	HR (95%CI)	1.00 (reference)	0.85 (0.56–1.28)	1.92 (1.08–3.40)	0.29
	RR (95%CI)	1.00 (reference)	0.79 (0.52–1.20)	1.72 (0.96–3.10)	0.56
Ischemic heart disease	n	20	11	5	
	Mortality rate (/1000 PY)	1.4	1.5	3.4	
	HR (95%CI)	1.00 (reference)	1.11 (0.53–2.31)	2.64 (0.99–7.04)	0.13
	RR (95%CI)	1.00 (reference)	1.06 (0.51–2.21)	2.27 (0.84–6.05)	0.24
Heart failure	n	42	19	5	
	Mortality rate (/1000 PY)	2.9	2.7	3.4	
	HR (95%CI)	1.00 (reference)	0.86 (0.50–1.48)	1.20 (0.47–3.04)	0.94
	RR (95%CI)	1.00 (reference)	0.82 (0.48–1.44)	1.07 (0.42–2.72)	0.76
Cerebrovascular disease	n	73	35	5	
	Mortality rate (/1000 PY)	5.0	4.9	3.4	
	HR (95%CI)	1.00 (reference)	0.98 (0.65–1.47)	0.75 (0.30–1.86)	0.63
	RR (95%CI)	1.00 (reference)	0.90 (0.60–1.36)	0.59 (0.24–1.46)	0.26
Cerebral hemorrhage	n	13	10	0	
	Mortality rate (/1000 PY)	0.90	1.4	0	
	HR (95%CI)	1.00 (reference)	1.51 (0.66–3.44)	-	0.95
	RR (95%CI)	1.00 (reference)	1.46 (0.64–3.36)	-	0.81
Cerebral infarction	n	42	14	2	
	Mortality rate (/1000 PY)	2.9	2.0	1.4	
	HR (95%CI)	1.00 (reference)	0.77 (0.42–1.41)	0.63 (0.15–2.60)	0.31
	RR (95%CI)	1.00 (reference)	0.63 (0.34–1.16)	0.42 (0.10–1.73)	0.07
Subarachnoidal hemorrhage	n	2	2	0	
	Mortality rate (/1000 PY)	0.14	0.28	0	
	HR (95%CI)	-	-	-	
	RR (95%CI)				
Women					
Circulatory disease	n	75	138	62	
	Mortality rate (/1000 PY)	8.3	9.1	8.9	
	HR (95%CI)	1.00 (reference)	1.30 (0.98–1.73)	1.33 (0.94–1.86)	0.08
	RR (95%CI)	1.00 (reference)	1.17 (0.87–1.57)	1.20 (0.85–1.71)	0.28
Cardiovascular disease	n	37	67	37	
	Mortality rate (/1000 PY)	4.1	4.4	5.3	
	HR (95%CI)	1.00 (reference)	1.36 (0.90–2.05)	1.71 (1.07–2.71)	0.02
	RR (95%CI)	1.00 (reference)	1.13 (0.74–1.71)	1.46 (0.91–2.35)	0.13
Ischemic heart disease	n	10	15	6	
	Mortality rate (/1000 PY)	1.10	0.99	0.86	
	HR (95%CI)	1.00 (reference)	1.07 (0.48–2.40)	0.97 (0.35–2.70)	0.98
	RR (95%CI)	1.00 (reference)	0.91 (0.41–2.05)	0.85 (0.31–2.34)	0.75
Heart failure	n	20	38	26	
	Mortality rate (/1000 PY)	2.2	2.5	3.7	
	HR (95%CI)	1.00 (reference)	1.53 (0.88–2.66)	2.37 (1.31–4.30)	0.004
	RR (95%CI)	1.00 (reference)	1.17 (0.67–2.05)	1.94 (1.06–3.54)	0.038
Cerebrovascular disease	n	34	69	25	
	Mortality rate (/1000 PY)	3.7	4.6	3.6	
	HR (95%CI)	1.00 (reference)	1.36 (0.90–2.07)	1.11 (0.66–1.87)	0.57
	RR (95%CI)	1.00 (reference)	1.23 (0.81–1.85)	1.00 (0.60–1.67)	0.89
Cerebral hemorrhage	n	6	20	2	
	Mortality rate (/1000 PY)	0.66	1.32	0.29	
	HR (95%CI)	1.00 (reference)	2.05 (0.82–5.11)	0.46 (0.09–2.26)	0.64
	RR (95%CI)	1.00 (reference)	1.98 (0.79–4.94)	0.44 (0.09–2.18)	0.47
Cerebral infarction	n	23	28	13	
	Mortality rate (/1000 PY)	2.5	1.8	1.9	
	HR (95%CI)	1.00 (reference)	0.87 (0.50–1.53)	0.92 (0.46–1.84)	0.77
	RR (95%CI)	1.00 (reference)	0.70 (0.40–1.21)	0.73 (0.37–1.45)	0.33
Subarachnoidal hemorrhage	n	2	8	4	
	Mortality rate (/1000 PY)	0.22	0.53	0.57	
	HR (95%CI)	1.00 (reference)	2.34 (0.54–11.0)	2.56 (0.47–14.0)	0.28
	RR (95%CI)	1.00 (reference)	2.38 (0.50–11.2)	2.59 (0.47–14.2)	0.23

β2-MG, β2-microglobulin; Cd, cadmium; n, number of deaths; PY, person-years; HR, age-adjusted hazard ratio calculated using the Cox proportional hazards model; RR, age-adjusted risk ratio calculated by the Fine and Gray competing risk regression model; CI, confidence interval.

**Table 5:** Risk of mortality from non-cancer/non-circulatory diseases according to the urinary Cd concentration in the participants with a urinary β2-MG level less than 1000 μg/g Cr.

		Urinary Cadmium Concentration (μg/g Cr)			*p* for Trend
		<5.0	5.0–9.9	≥10.0	
Men					
Endocrine, nutritional, and metabolic diseases	n	8	6	0	
	Mortality rate (/1000 PY)	0.55	0.84	0	
	HR (95%CI)	1.00 (reference)	1.53 (0.53–4.41)	-	0.98
	RR (95%CI)	1.00 (reference)	1.50 (0.52–4.32)	-	0.89
Diseases of the respiratory system	n	95	53	14	
	Mortality rate (/1000 PY)	6.5	7.5	9.5	
	HR (95%CI)	1.00 (reference)	1.14 (0.81–1.59)	1.63 (0.93–2.85)	0.11
	RR (95%CI)	1.00 (reference)	1.08 (0.77–1.52)	1.36 (0.77–2.42)	0.32
Pneumonia and influenza	n	59	26	11	
	Mortality rate (/1000 PY)	4.1	3.7	7.5	
	HR (95%CI)	1.00 (reference)	0.92 (0.58–1.46)	2.10 (1.10–4.00)	0.19
	RR (95%CI)	1.00 (reference)	0.83 (0.52–1.33)	1.73 (0.90–3.31)	0.51
Diseases of the digestive system	n	16	11	6	
	Mortality rate (/1000 PY)	1.1	1.5	4.1	
	HR (95%CI)	1.00 (reference)	1.40 (0.65–3.03)	3.81 (1.49–9.74)	0.014
	RR (95%CI)	1.00 (reference)	1.37 (0.63–2.96)	3.52 (1.39–8.95)	0.028
Liver cirrhosis	n	5	5	1	
	Mortality rate (/1000 PY)	0.34	0.70	0.68	
	HR (95%CI)	1.00 (reference)	2.05 (0.59–7.09)	1.91 (0.22–16.3)	0.68
	RR (95%CI)	1.00 (reference)	2.04 (0.60–6.95)	1.87 (0.22–15.9)	0.25
Kidney and urinary tract diseases	n	14	6	2	
	Mortality rate (/1000 PY)	0.96	0.84	1.36	
	HR (95%CI)	1.00 (reference)	0.83 (0.32–2.17)	1.67 (0.38–7.39)	0.81
	RR (95%CI)	1.00 (reference)	0.80 (0.31–2.10)	1.27 (0.29–5.56)	0.96
Senility	n	20	17	2	
	Mortality rate (/1000 PY)	1.4	2.4	1.4	
	HR (95%CI)	1.00 (reference)	1.64 (0.85–3.16)	1.17 (0.27–5.05)	0.27
	RR (95%CI)	1.00 (reference)	1.60 (0.83–3.08)	0.88 (0.20–3.80)	0.40
External causes of mortality	n	31	18	4	
	Mortality rate (/1000 PY)	2.1	2.5	2.7	
	HR (95%CI)	1.00 (reference)	1.19 (0.67–2.13)	1.33 (0.47–3.77)	0.47
	RR (95%CI)	1.00 (reference)	1.14 (0.64–2.04)	1.19 (0.41–3.39)	0.62
Women					
Endocrine, nutritional, and metabolic diseases	n	2	12	4	
	Mortality rate (/1000 PY)	0.22	0.79	0.57	
	HR (95%CI)	1.00 (reference)	3.72 (0.83–16.7)	2.79 (0.51–15.3)	0.25
	RR (95%CI)	1.00 (reference)	3.46 (0.78–15.4)	2.58 (0.47–14.2)	0.20
Diseases of the respiratory system	n	35	50	28	
	Mortality rate (/1000 PY)	3.9	3.3	4.0	
	HR (95%CI)	1.00 (reference)	1.06 (0.68–1.65)	1.37 (0.83–2.27)	0.24
	RR (95%CI)	1.00 (reference)	0.82 (0.53–1.27)	1.08 (0.65–1.78)	0.87
Pneumonia and influenza	n	18	36	22	
	Mortality rate (/1000 PY)	2.0	2.4	3.2	
	HR (95%CI)	1.00 (reference)	1.55 (0.87–2.77)	2.22 (1.17–4.19)	0.014
	RR (95%CI)	1.00 (reference)	1.17 (0.66–2.05)	1.68 (0.90–3.13)	0.11
Diseases of the digestive system	n	9	13	11	
	Mortality rate (/1000 PY)	0.99	0.86	1.6	
	HR (95%CI)	1.00 (reference)	1.02 (0.43–2.41)	1.95 (0.80–4.74)	0.15
	RR (95%CI)	1.00 (reference)	0.87 (0.37–2.03)	1.71 (0.71–4.10)	0.28
Liver cirrhosis	n	2	3	3	
	Mortality rate (/1000 PY)	0.22	0.20	0.43	
	HR (95%CI)	-	-	-	
	RR (95%CI)				
Kidney and urinary tract diseases	n	4	12	4	
	Mortality rate (/1000 PY)	0.44	0.79	0.57	
	HR (95%CI)	1.00 (reference)	2.07 (0.66–6.52)	1.56 (0.38–6.33)	0.49
	RR (95%CI)	1.00 (reference)	1.75 (0.56–5.44)	1.33 (0.33–5.41)	0.61
Senility	n	22	38	11	
	Mortality rate (/1000 PY)	2.4	2.5	1.6	
	HR (95%CI)	1.00 (reference)	1.38 (0.80–2.36)	0.93 (0.45–1.95)	0.93
	RR (95%CI)	1.00 (reference)	1.04 (0.61–1.77)	0.70 (0.33–1.45)	0.38
External causes of mortality	n	7	17	13	
	Mortality rate (/1000 PY)	0.77	1.1	1.9	
	HR (95%CI)	1.00 (reference)	1.47 (0.61–3.56)	2.51 (1.00–6.30)	0.044
	RR (95%CI)	1.00 (reference)	1.41 (0.59–3.40)	2.44 (0.97–6.16)	0.057

β2-MG, β2-microglobulin; Cd, cadmium; n, number of deaths; PY, person-years; HR, age-adjusted hazard ratio calculated using the Cox proportional hazards model; RR, age-adjusted risk ratio calculated by the Fine and Gray competing risk regression model; CI, confidence interval.

**Table 6:** Risks of cause-specific mortality according to the urinary Cd concentration in the participants with a urinary β2-MG level of 1000 μg/g Cr or higher.

		Urinary Cd Concentration (μg/g Cr)			*p* for Trend
		<5.0	5.0–9.9	≥10.0	
Men					
Malignant neoplasms	n	7	14	17	
	Mortality rate (/1000 PY)	12.7	18.8	20.1	
	HR (95%CI)	1.00 (reference)	1.22 (0.48–3.10)	1.30 (0.53–3.17)	0.58
	RR (95%CI)	1.00 (reference)	1.34 (0.56–3.21)	1.55 (0.66–3.64)	0.31
Endocrine, nutritional, and metabolic diseases	n	0	2	0	
	Mortality rate (/1000 PY)	0	2.7	0	
Circulatory disease	n	19	23	26	
	Mortality rate (/1000 PY)	34.6	30.9	30.7	
	HR (95%CI)	1.00 (reference)	0.66 (0.36–1.23)	0.74 (0.41–1.34)	0.40
	RR (95%CI)	1.00 (reference)	0.61 (0.32–1.15)	0.72 (0.40–1.32)	0.40
Cardiovascular disease	n	10	7	14	
	Mortality rate (/1000 PY)	18.2	9.4	16.5	
	HR (95%CI)	1.00 (reference)	0.37 (0.14–0.99)	0.71 (0.31–1.62)	0.62
	RR (95%CI)	1.00 (reference)	0.36 (0.13–0.98)	0.79 (0.35–1.78)	0.82
Heart failure	n	4	4	7	
	Mortality rate (/1000 PY)	7.3	5.4	8.3	
	HR (95%CI)	1.00 (reference)	0.48 (0.12–1.95)	0.87 (0.25–3.02)	0.99
	RR (95%CI)	1.00 (reference)	0.51 (0.13–2.01)	1.00 (0.29–3.43)	0.85
Cerebrovascular disease	n	9	12	11	
	Mortality rate (/1000 PY)	16.4	16.1	13.0	
	HR (95%CI)	1.00 (reference)	0.74 (0.31–1.77)	0.66 (0.27–1.60)	0.38
	RR (95%CI)	1.00 (reference)	0.73 (0.30–1.76)	0.69 (0.28–1.67)	0.45
Diseases of the respiratory system	n	5	8	11	
	Mortality rate (/1000 PY)	9.1	10.7	13.0	
	HR (95%CI)	1.00 (reference)	0.99 (0.31–3.13)	1.25 (0.43–3.65)	0.63
	RR (95%CI)	1.00 (reference)	1.02 (0.30–3.43)	1.38 (0.46–4.17)	0.52
Pneumonia and influenza	n	2	3	7	
	Mortality rate (/1000 PY)	3.6	4.0	8.3	
	HR (95%CI)	1.00 (reference)	0.87 (0.14–5.30)	1.90 (0.39–9.27)	0.30
	RR (95%CI)	1.00 (reference)	0.99 (0.14–6.98)	2.26 (0.43–11.7)	0.25
Diseases of the digestive system	n	1	6	2	
	Mortality rate (/1000 PY)	1.8	8.1	2.4	
Kidney and urinary tract diseases	n	2	7	4	
	Mortality rate (/1000 PY)	3.6	9.4	4.7	
	HR (95%CI)	1.00 (reference)	2.00 (0.41–9.83)	1.02 (0.19–5.68)	0.84
	RR (95%CI)	1.00 (reference)	2.41 (0.48–12.2)	1.24 (0.23–6.64)	1.00
Senility	n	3	6	5	
	Mortality rate (/1000 PY)	5.5	8.1	5.9	
	HR (95%CI)	1.00 (reference)	0.68 (0.16–2.91)	0.87 (0.20–3.83)	0.94
	RR (95%CI)	1.00 (reference)	0.73 (0.20–2.69)	0.90 (0.22–3.59)	0.96
Women					
Malignant neoplasms	n	1	11	26	
	Mortality rate (/1000 PY)	4.4	8.4	9.8	
	HR (95%CI)	1.00 (reference)	1.94 (0.25–15.1)	2.25 (0.30–16.9)	0.42
	RR (95%CI)	1.00 (reference)	2.46 (0.32–19.2)	2.91 (0.38–22.3)	0.29
Endocrine, nutritional, and metabolic diseases	n	0	2	5	
	Mortality rate (/1000 PY)	0	1.5	1.9	
Circulatory disease	n	9	44	57	
	Mortality rate (/1000 PY)	39.8	33.6	21.5	
	HR (95%CI)	1.00 (reference)	1.02 (0.50–2.10)	0.75 (0.37–1.53)	0.16
	RR (95%CI)	1.00 (reference)	1.10 (0.52–2.29)	0.74 (0.36–1.52)	0.083
Cardiovascular disease	n	4	26	31	
	Mortality rate (/1000 PY)	17.7	19.9	11.7	
	HR (95%CI)	1.00 (reference)	1.28 (0.44–3.68)	0.86 (0.30–2.46)	0.27
	RR (95%CI)	1.00 (reference)	1.55 (0.54–4.44)	0.96 (0.34–2.70)	0.23
Heart failure	n	2	17	19	
	Mortality rate (/1000 PY)	8.8	13.0	7.2	
	HR (95%CI)	1.00 (reference)	1.76 (0.40–7.65)	1.16 (0.27–5.01)	0.52
	RR (95%CI)	1.00 (reference)	2.09 (0.50–8.78)	1.28 (0.30–5.34)	0.47
Cerebrovascular disease	n	5	18	23	
	Mortality rate (/1000 PY)	22.1	13.8	8.7	
	HR (95%CI)	1.00 (reference)	0.81 (0.30–2.18)	0.59 (0.22–1.55)	0.19
	RR (95%CI)	1.00 (reference)	0.81 (0.29–2.24)	0.57 (0.21–1.53)	0.16
Diseases of the respiratory system	n	4	5	13	
	Mortality rate (/1000 PY)	17.7	3.8	4.9	
	HR (95%CI)	1.00 (reference)	0.24 (0.07–0.92)	0.34 (0.11–1.07)	0.30
	RR (95%CI)	1.00 (reference)	0.26 (0.07–1.00)	0.37 (0.11–1.20)	0.42
Pneumonia and influenza	n	3	4	9	
	Mortality rate (/1000 PY)	13.3	3.1	3.4	
	HR (95%CI)	1.00 (reference)	0.28 (0.06–1.28)	0.34 (0.09–1.31)	0.31
	RR (95%CI)	1.00 (reference)	0.29 (0.07–1.28)	0.36 (0.10–1.37)	0.41
Diseases of the digestive system	n	2	7	8	
	Mortality rate (/1000 PY)	8.8	5.3	3.0	
	HR (95%CI)	1.00 (reference)	0.69 (0.14–3.36)	0.47 (0.10–2.21)	0.28
	RR (95%CI)	1.00 (reference)	0.81 (0.17–3.90)	0.50 (0.11–2.42)	0.26
Kidney and urinary tract diseases	n	0	4	11	
	Mortality rate (/1000 PY)	0	3.1	4.2	
Senility	n	6	11	27	
	Mortality rate (/1000 PY)	26.5	8.4	10.2	
	HR (95%CI)	1.00 (reference)	0.42 (0.15–1.13)	0.56 (0.23–1.37)	0.62
	RR (95%CI)	1.00 (reference)	0.36 (0.13–1.03)	0.64 (0.26–1.58)	0.90

β2-MG, β2-microglobulin; Cd, cadmium; n, number of deaths; PY, person-years; HR, age-adjusted hazard ratio calculated using the Cox proportional hazards model; RR, age-adjusted risk ratio calculated by the Fine and Gray competing risk regression model; CI, confidence interval.

## Data Availability

The data that supports the findings of this study are available from the corresponding author, upon reasonable request.
